# Impact of phylogeny on structural contact inference from protein sequence data

**DOI:** 10.1098/rsif.2022.0707

**Published:** 2023-02-08

**Authors:** Nicola Dietler, Umberto Lupo, Anne-Florence Bitbol

**Affiliations:** ^1^ Institute of Bioengineering, School of Life Sciences, École Polytechnique Fédérale de Lausanne (EPFL), 1015 Lausanne, Switzerland; ^2^ SIB Swiss Institute of Bioinformatics, 1015 Lausanne, Switzerland

**Keywords:** protein sequences, inference, contact prediction, phylogeny, modelling, data analysis

## Abstract

Local and global inference methods have been developed to infer structural contacts from multiple sequence alignments of homologous proteins. They rely on correlations in amino acid usage at contacting sites. Because homologous proteins share a common ancestry, their sequences also feature phylogenetic correlations, which can impair contact inference. We investigate this effect by generating controlled synthetic data from a minimal model where the importance of contacts and of phylogeny can be tuned. We demonstrate that global inference methods, specifically Potts models, are more resilient to phylogenetic correlations than local methods, based on covariance or mutual information. This holds whether or not phylogenetic corrections are used, and may explain the success of global methods. We analyse the roles of selection strength and of phylogenetic relatedness. We show that sites that mutate early in the phylogeny yield false positive contacts. We consider natural data and realistic synthetic data, and our findings generalize to these cases. Our results highlight the impact of phylogeny on contact prediction from protein sequences and illustrate the interplay between the rich structure of biological data and inference.

## 1. Introduction

Statistical inference from biological data is currently making great advances, thanks to the rapid growth of available data, and to the development and application of inference methods. These methods range from interpretable models inspired by statistical physics to deep learning approaches. In this exciting context, it is important to understand the impact of the rich and complex structure of biological data on the performance of inference methods [1].

In particular, progress in sequencing has caused a spectacular growth of available genome sequences. This allows inference to be performed on large multiple sequence alignments (MSAs) of families of homologous proteins. Because proteins in the same family share the same ancestry, as well as similar three-dimensional structures and functional properties, these MSA data are highly structured. In particular, MSAs comprise correlations coming from phylogeny [2–4], as well as from structural and from functional constraints [5,6]. Pairwise maximum entropy models [7,8], known as Potts models or direct coupling analysis (DCA) [9], trained on natural MSAs, have revealed structural contacts [10–12]. They have also been employed to analyse mutational effects [13–16], protein evolution [17] and conformational changes [18,19], to design proteins [20], as well as to predict interaction partners among paralogs [21,22] and protein–protein interaction networks [23,24]. The key idea of these fruitful approaches is coevolution: amino acids that are in contact in the three-dimensional structure of proteins need to maintain physico-chemical complementarity, yielding correlations in amino acid usage at contacting sites. Recently, AlphaFold, a deep learning approach exploiting the breadth of available sequences and experimentally determined structures, has brought major advances to the computational prediction of protein structures from sequences [25]. Despite the important methodological differences with DCA, coevolution between amino acids is also an important ingredient of AlphaFold, which starts by constructing an MSA of homologues when given a protein sequence as input [25,26].

How does the complex structure of MSA data impact the performance of statistical inference? In MSAs, correlations due to phylogeny [2,4] and due to selection to preserve structure [10–12] and function coexist. Thus, phylogenetic correlations can impair the inference of structural contacts. This effect has been demonstrated both when using mutual information (MI) [27] and when using inferred Potts models [4,28–30]. It has motivated the development and use of various empirical corrections aimed at reducing the impact of phylogenetic correlations [27,31], such as the average product correction (APC) [32,33], phylogenetic reweighting [9–11,33,34] and nested coevolution [35].

How do phylogenetic correlations impact MSA properties and impair contact prediction using MI, covariance or inferred Potts models? To address this question, we generate synthetic data from a minimal model where the amount of correlations from structural contacts and from phylogeny can be fully controlled. We analyse the impact of the strength of natural selection to preserve structure, the impact of phylogenetic relatedness and their interplay. While phylogeny impairs contact prediction, we show that Potts models are more robust to phylogeny than local scores, even in the absence of explicit phylogenetic corrections, which are useful in both cases. This robustness to phylogeny comes on top of the ability of Potts models to disentangle direct and indirect correlations (which only matters for strong selection), and may explain the success of these methods. We further show that sites that mutate early in the phylogeny yield false positive (FP) contacts. Next, we analyse natural protein sequence data and realistic synthetic data generated from models inferred on natural data, either with or without phylogeny. Our findings from the minimal model generalize well to these cases.

## 2. Results

### 2.1. Minimal model with structural constraints and phylogeny

**Figure 1.**
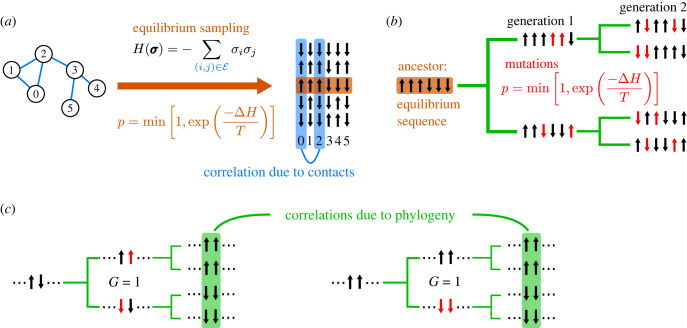
Data generation and origins of correlations. (*a*) In our model, couplings between nodes are set to 1 on the edges of an Erdös–Rényi random graph, and to 0 otherwise (the set of edges is denoted by E). Placing a spin *σ*_*i*_ on each node of the graph gives rise to the Hamiltonian *H* for a sequence σ of spins. Equilibrium sampling of sequences (without phylogeny) is performed using a Metropolis Monte Carlo algorithm, with move acceptance probability *p* defined as shown, starting from random initial sequences. An example correlation arising from a coupling is shown between columns 0 and 2 in the resulting set of independently generated equilibrium sequences (blue). (*b*) To generate sequences with phylogeny, an equilibrium sequence (generated as in (*a*)) is taken as the ancestor, and is evolved along a binary branching tree (green) where mutations (red) are accepted with probability *p*. On each branch of the tree, *μ* mutations are accepted, with *μ* = 2 here. (*c*) Examples of phylogenetic correlations. On the left-hand side, early mutations arise at two sites in different sequences, leading to highly correlated sites in their daughter sequences (assuming no further mutations at those sites). On the right-hand side, early mutations arise simultaneously in one sequence, also giving a large correlation between these sites in the daughter sequences. In both cases, the earliest mutation generation *G* of the pair of sites of interest is given. It is defined as the first generation in the tree at which both sites have mutated with respect to their state in the ancestral sequence.

Separating correlations from structural contacts and from phylogeny is extremely tricky in natural data [36]. Thus, to assess the impact of phylogeny on structural contact prediction, we generate and study synthetic data with controlled amounts of correlations from structural constraints and from phylogeny. For this, we consider a minimal model, where protein sequences are represented by sequences of Ising spins, each spin sitting on one node of a fixed Erdös–Rényi random graph. Structural constraints, representing contacts in the three-dimensional protein structure, are modelled by pairwise couplings on the edges of this graph. We then sample independent equilibrium sequences using a Metropolis–Hastings algorithm (see Models and methods and electronic supplementary material, figure S1). This yields datasets of sequences that are only subject to the structural constraints defined by the graph. These datasets are regarded as MSAs. All correlations between sites then arise from these constraints or from finite-size effects. In order to introduce phylogeny in a controlled way, we start from one equilibrium sequence, considered as the ancestral sequence, and we evolve sequences on a binary branching tree with a fixed number *μ* of accepted mutations, i.e. substitutions, per branch. Mutations are accepted using the same Metropolis criterion as when generating independent equilibrium sequences. The sequences at the leaves of the tree yield an MSA that incorporates structural constraints and phylogeny. Both of them can yield correlations between the columns of the MSA. Our method to generate data is illustrated in figure 1, and described in detail in Models and methods. Note that the case with phylogeny and no selection is a limiting case of our model (see below).

### 2.2. Impact of phylogeny on contact prediction

**Figure 2.**
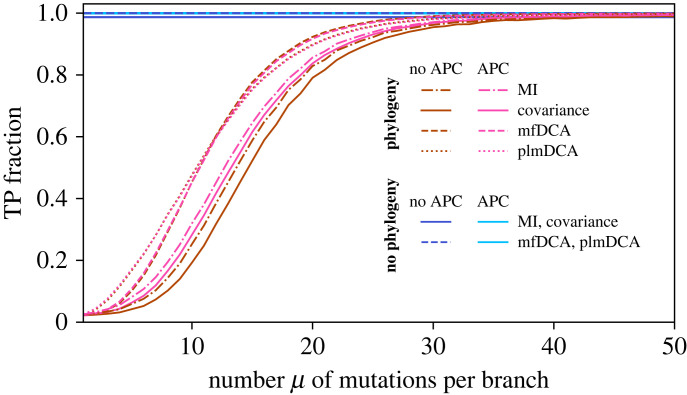
Impact of phylogeny on contact prediction. The fraction of correctly predicted contacts (true positive (TP) fraction) is shown versus the number *μ* of accepted mutations (i.e. substitutions) per branch of the phylogenetic tree. Four different inference methods are compared: covariance, MI, mfDCA and plmDCA. In all cases, results are presented without and with APC. The data generated without phylogeny, used for reference, comprises *M* = 2048 sequences of length ℓ = 200 sampled at equilibrium by Metropolis Monte Carlo at *T* = 5 under the Hamiltonian in equation (4.1) on an Erdös–Rényi graph with edge probability *q* = 0.02, representing the contact map. Data with phylogeny is generated starting from one of the equilibrium sequences, and this ‘ancestor’ is evolved along a binary branching tree, with a fixed number *μ* of accepted mutations per branch, for 11 generations, yielding *M* = 2^11^ = 2048 sequences. Random proposed mutations (spin flips) are accepted according to the Metropolis criterion at *T* = 5 under the Hamiltonian in equation (4.1) on the same Erdös–Rényi graph. All results are averaged over 100 realizations (i.e. 100 data generations, always for the same Erdös–Rényi graph). Note that the standard deviation of the TP fraction is always smaller than 10^−2^. Note also that inference with APC on the data generated without phylogeny is as good for local and global methods, so they are shown with the same linestyle and colour.

What is the impact of phylogeny on contact prediction in this minimal model? Varying *μ* allows us to tune the amount of phylogeny. A small *μ* means that sequences are very closely related, while independent equilibrium sequences are recovered in the limit *μ* → ∞. In figure 2, we show the performance of contact prediction versus *μ*. This performance is quantified by the TP fraction of predicted contacts, evaluated at the number of edges in the graph (see Models and methods), and we compare it with that obtained for equilibrium datasets. We perform contact inference with four different methods: two local ones, based on covariance and MI between sites, and two global ones, based on inferring DCA Potts models that fit the one- and two-body frequencies of the data. Local methods directly compare pairwise statistics, while global methods aim to infer a probability distribution for the whole sequence. The two global methods, mean-field DCA (mfDCA) [10,11] and pseudolikelihood maximization DCA (plmDCA) [32,37] (see Models and methods), employ different approximation schemes in the Potts model inference. In all cases, we present results both with and without the APC, which was introduced to correct for biases due to conservation and phylogeny when using MI to predict structural contacts [27]. This correction improves contact inference performance by DCA on natural protein sequence data [32,37], and is widely used. Furthermore, we find that it improves contact prediction performance substantially more than phylogenetic reweighting when using synthetic data generated from models inferred on natural data (see below). For equilibrium data, all inference methods yield a very good performance, with TP fractions close to 1. For data generated with phylogeny, the equilibrium results are recovered for large *μ*, as expected. However, inference performance is substantially impaired for smaller values of *μ* (corresponding to strong phylogeny). Importantly, global (DCA) methods are more resilient to phylogenetic noise than local ones (covariance and MI) for small and intermediate values of *μ*. This result is robust to whether APC is used or not, but we note that APC improves the performance of local methods when phylogeny is strong, while it has almost no effect on the performance of global methods. For instance, for *μ* = 15, the TP fraction obtained by plmDCA is 28% (resp. 17%) higher than that obtained by MI without (resp. with) APC. APC generally improves the performance of both local [27] and global methods [32,37] on natural sequence data (see also our results below on natural and more realistic data). The success of APC with global methods may then be partly due to its ability to correct for conservation effects [27], corresponding to non-zero fields in Potts models. While, in our minimal model, there is a sharp separation between non-contacts and contacts, inferred DCA models comprise many small non-zero couplings, due to phylogeny, functional constraints and finite-size effects. Our result that DCA methods are more robust to phylogeny than local methods still holds when a background of non-zero couplings is included (see electronic supplementary material, figure S2).

### 2.3. Impact of selection strength on contact prediction without phylogeny

**Figure 3.**
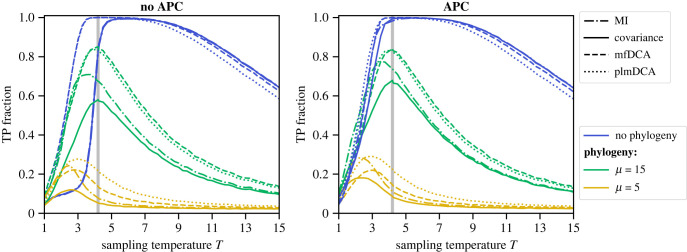
Impact of sampling temperature on contact prediction. The TP fraction is shown versus the Monte Carlo sampling temperature *T* for four different inference methods (covariance, MI, mfDCA, plmDCA). The left panel shows results without APC, and the right panel with APC. Three cases are considered: equilibrium data (no phylogeny), and numbers of accepted mutations per branch *μ* = 15 and *μ* = 5 (increasing impact of phylogeny). Data are generated as in figure 2, using the same contact map (Erdös–Rényi graph). The grey vertical line shows the approximate phase transition temperature *T*_*C*_. All results are averaged over 100 realizations.

In addition to *μ*, another important parameter of our model is the Monte Carlo sampling temperature *T* (see figure 1 and equation (4.2)). It plays the role of an inverse selection strength, where selection aims to preserve structure. Note that other selective pressures are not considered in our minimal model. For large *T*, almost all mutations are accepted, and structural constraints are weak. Therefore, in the limit *T* → ∞, selection vanishes and all correlations are from phylogeny. Conversely, for small *T*, essentially only the mutations that decrease the energy of the sequence of spins are accepted, and structural constraints become stringent. Figure 3 shows the performance of contact prediction versus *T* for equilibrium sequences (no phylogeny), and for sequences generated with *μ* = 15 and *μ* = 5. At equilibrium, our model possesses a ferromagnetic–paramagnetic phase transition, whose approximate temperature *T*_*C*_ = 4.2 was found by inspecting magnetization histograms [38]. For equilibrium sequences at low *T*, inference performance without APC is poor (figure 3, left panel), and DCA methods perform substantially better than local methods (which are overlapping). The performance of DCA methods increases with *T*, and reaches a perfect score before *T*_*C*_. Without APC, local methods also reach a perfect contact prediction score, but after *T*_*C*_. However, they perform slightly better than DCA at higher temperatures. These trends are consistent with those previously described in [1,38], which analysed synthetic data generated independently at equilibrium, i.e. without phylogeny. Inference is difficult either if sequences are all frozen and redundant (very small *T*) or if sequences are too noisy (very large *T*), yielding better performance for intermediate values of *T*. Furthermore, APC substantially improves the performance of local methods at equilibrium, and makes their performance comparable to that of global methods even at small *T* (figure 3, right panel). In electronic supplementary material, section S3, we show that the weak performance of local methods on equilibrium data at low *T* and without APC is associated with conservation properties (see electronic supplementary material, figure S3), partly mitigated by regularization in global methods. Furthermore, in electronic supplementary material, section S4, we present a detailed comparison with [1], which shows that our results are fully consistent with theirs, and that regularization is crucial to the performance of DCA (see electronic supplementary material, figure S4).

### 2.4. Interplay between phylogeny and selection strength

What is the impact of phylogeny on the temperature dependence of contact prediction performance? As for equilibrium data, the best performance is obtained for intermediate values of *T* with phylogenetic data (see figure 3). However, the maximum of performance shifts to lower values of *T* when phylogeny is increased. This may be because, when phylogeny is strong, stringent selection (low *T*) keeps contacts relevant, thereby compensating for phylogeny, while at high *T*, the combination of thermal noise and phylogeny makes contact inference particularly tricky. Moreover, with phylogeny, DCA methods yield substantially better results than local methods for intermediate to high values of *T*, by contrast with the equilibrium case. This holds whether or not APC is used, even though APC improves the performance of local methods. Note, however, that MI slightly outperforms DCA methods at small values of *T*. Overall, the fact that DCA outperforms local methods in a wide range of selection strengths in the presence of phylogeny (generalizing the result observed at *T* = 5 in figure 2) is much better in line with observations on natural data [11] than with results obtained without phylogeny, where DCA yields at best marginal improvement compared with local methods with APC. Thus, robustness to phylogeny may be what makes DCA superior to local methods. We also note that plmDCA outperforms mfDCA when phylogeny is strongest (*μ* = 5), which is reminiscent of the results obtained on natural data [32]. In electronic supplementary material, section S4, we consider a denser Erdös–Rényi graph, and our results are robust to this change (see electronic supplementary material, figures S4 and S5). However, larger values of pseudocounts or regularization strength than the usual ones are then helpful (see also electronic supplementary material, figure S6). The contact density used throughout and this denser one are both in the range observed in natural proteins (see electronic supplementary material, table S1).

### 2.5. Impact of graph properties on contact inference

Before exploring in more detail the impact of phylogeny, let us analyse the impact of graph properties, reflecting structural constraints, on equilibrium results. Indeed, in phylogenetic data, this structural signal needs to be disentangled from phylogenetic correlations. For each pair of sites, we consider the shortest path length *L* in the graph connecting the two sites—*L* = 1 denotes contacting sites. Normalized histograms of coevolution scores for all pairs of sites are shown versus *L* in electronic supplementary material, figure S7 without APC and in electronic supplementary material, figure S8 with APC. For *T* = 3 < *T*_*C*_, without APC and especially for local methods, histograms of pairs in contact (*L* = 1) have substantial overlap with other histograms, especially with those for *L* = 2, showing that indirect correlations impair inference. However, APC strongly mitigates this issue, allowing local methods to perform almost as well as global ones, as seen in figure 3. Close to *T*_*C*_, histograms of coevolution scores feature no overlap for DCA methods, leading to perfect inference. The small overlaps that still exist for local methods are resolved by using APC. Thus, APC enables local methods to disentangle direct and indirect correlations at equilibrium almost as well as global ones. At *T* = 5 > *T*_*C*_, all methods yield perfect inference, as indirect correlations become weaker in the paramagnetic phase. Note also that plmDCA performs better than mfDCA at separating pairs with *L* = 1 and *L* = 2.

In addition to *L*, we consider another descriptor of graph structure, namely the number *N* of nearest neighbours of each site. To assess the impact of *N* on the variance of equilibrium data, we perform principal component analysis (PCA) focusing on the matrix of covariances between sequences [2]. Electronic supplementary material, figure S9 shows the coordinates of sites on the first principal component versus *N*. At all temperatures considered, and most strongly at low ones, these coordinates are correlated with *N*, showing that connectivity is a crucial ingredient of the variance of sites. Indeed, groups of connected spins tend to be aligned together at low temperatures, while isolated spins are independent. This difference is strongest at low *T* (see equation (4.2)).

### 2.6. Origin and impact of phylogenetic correlations

**Figure 4.**
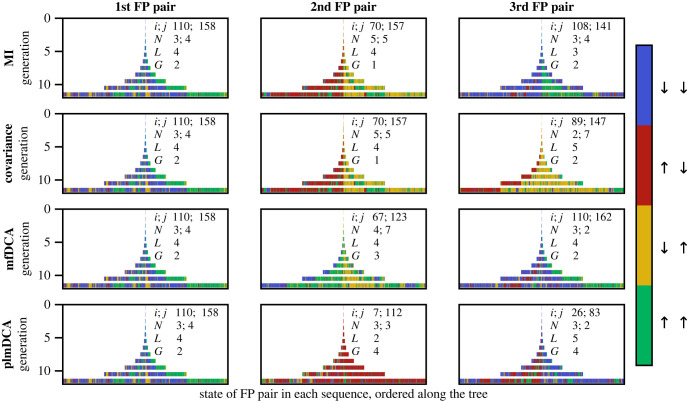
Evolution of false positive (FP) pairs along the phylogenetic tree. For each inference method (MI, covariance, mfDCA, plmDCA), the state of the top three FP pairs of sites (*i*, *j*), wrongly inferred as contacts from the evolved sequences, is depicted along the phylogenetic tree. This allows to track the history of these pairs (*i*, *j*). At each generation, each vertical bar represents one sequence, and the colour of the bar indicates the state of the FP pair of focus within this sequence. Hence, the ancestor sequence is the single bar at generation 0, and the final generation (11) contains 2048 bars corresponding to all evolved sequences. Data are generated as in figure 2, using the same contact map (Erdös–Rényi graph), at *T* = 5 and *μ* = 5 (strong phylogeny). Characteristics of each FP pair (*i*, *j*) are reported, namely the indices of the sites *i* and *j*, their number *N* of neighbours in the graph (i.e. sites in contact with *i* or *j*), the length *L* of the shortest path connecting *i* to *j* in the graph (*L* = 1 for contacts), and the earliest mutation generation *G* where both *i* and *j* have mutated with respect to the ancestral sequence.

How does phylogeny impair contact inference? What pairs of sites appear to be coupled due to phylogeny, while they are actually not coupled? To address these questions, we focus on the FP pairs which have the highest scores. Our synthetic data make it possible to investigate the states of these pairs of sites through the phylogenetic tree, starting from the ancestral sequence. The states of the top three FP pairs found by each method are shown in figure 4 for a dataset generated at *μ* = 5 and *T* = 5 (i.e. with strong phylogeny). We observe large blocks of colours in our representation, meaning that these pairs of sites underwent mutations early in the phylogeny, leading to strongly correlated sites in the last generation. Indeed, early mutations at two sites produce correlations among them, as illustrated in figure 1*b*. This effect can be quantified by attributing to each pair of sites an index *G*, which is the first generation at which each of the spins of this pair has flipped (i.e. mutated), in at least one of the sequences generated thus far, with respect to its state in the ancestral sequence (see figure 1*b*). All top FP pairs in figure 4 have small *G* values, and this effect is even stronger for local methods (G=1 or 2) than for DCA methods (*G* = 2–4). Thus, pairs of sites mutating early in the phylogeny are more likely to have high coevolution scores due to phylogeny. Their shortest path length *L* is also indicative on the phylogenetic origin of their high coevolution scores. Indeed, *L* > 2 for all of them but one, while if indirect correlations were the main cause of these FPs, we would expect more occurrences of *L* = 2. Note that, in [39], we showed that these pairs of sites are particularly useful for the inference of interacting partners among the paralogs of two protein families, where phylogenetic correlations are useful.

**Figure 5.**
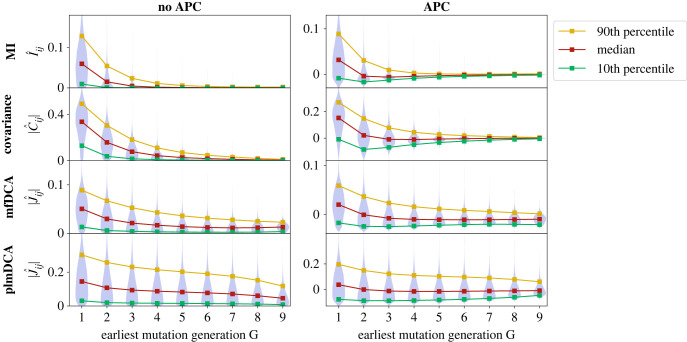
Impact of phylogeny on coevolution scores. Violin plots of the coevolution scores of all pairs of sites (*i*, *j*), inferred by four different methods (MI, covariance, mfDCA, plmDCA), are shown versus the earliest generation *G* where both *i* and *j* have mutated with respect to the ancestral sequence. The left panels show the raw Frobenius norm scores (absolute value here) and the right panels show these scores corrected with APC. Data are generated as in figure 2, using the same contact map (Erdös–Rényi graph), at *T* = 5 and *μ* = 5. Couplings were inferred on 100 datasets of 2048 sequences each, and aggregated.

How do these observations made on a few top FPs generalize? To assess this, we compute the index *G* of all pairs of sites (*i*, *j*)—including both contacts and non-contacts—in multiple synthetic datasets generated at *μ* = 5 and *T* = 5. (Note that the results that follow also hold when restricting to all FPs or to all non-contacts, which are much more numerous than contacts.) We then group pairs of sites that have a given value of *G*. Figure 5 shows the distribution of coevolution scores versus the index *G* as a violin plot (i.e. Gaussian kernel-smoothed vertical histograms). It sheds light on an overall dependence of the coevolution scores on *G*, with larger coevolution scores observed for small *G*, i.e. early mutations. Moreover, this dependence is weaker for global methods than for local ones, and among global methods, it is weaker for plmDCA than for mfDCA. These conclusions hold both without and with APC. This confirms that DCA, especially plmDCA, is more robust to phylogenetic correlations than local inference methods, which could partly explain its success on natural protein sequences. The behaviour of the median score versus *G* (red curve in figure 5) is further investigated in electronic supplementary material, figure S10. Without APC, the median decays abruptly with *G*, especially for local methods. This decrease is less abrupt and closer to linear for plmDCA, confirming its lower sensitivity to the phylogeny. APC substantially reduces the median for small *G* for all methods, thereby mitigating the impact of phylogeny on inference performance.

Besides, electronic supplementary material, figure S11 shows the average conservation of sites involved in a pair versus *G*. It shows that pairs of sites undergoing mutations early in the phylogeny are often weakly conserved. Qualitatively, sites with early mutations will often be in different states (up and down) in sequences at the leaves of the tree, yielding a poor conservation but a strong correlation. This point could be exploited to disentangle TP and FP pairs when the underlying phylogeny is not known. The inferred fields could also be useful to this end. However, we expect the general case to be more complex as conservation can arise both from single-site constraints modelled by non-zero fields and from late divergences along the phylogeny. Note that in sector analysis, the focus is on conserved correlations, thus exploiting both aspects [3,40].

### 2.7. Extension to more realistic synthetic data, generated from models inferred on natural data

**Figure 6.**
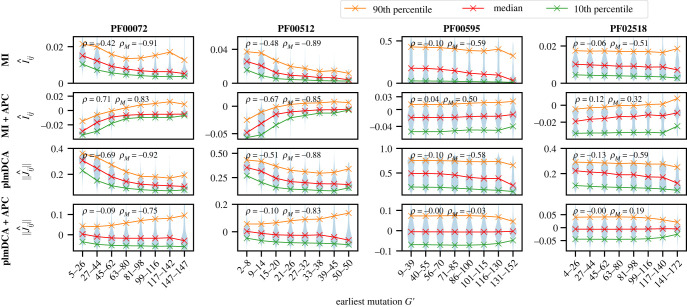
Impact of phylogeny on coevolution scores for more realistic data. Violin plots of the coevolution scores of all pairs of sites (*i*, *j*), inferred by MI or plmDCA, without or with APC, are shown versus the earliest mutation *G*′ where both *i* and *j* have mutated with respect to the ancestral sequence. To determine *G*′, we count the number *G*_*i*_ (resp. *G*_*j*_) of mutations undergone by a sequence when following the branches of the phylogenetic tree from the ancestral sequence to the first one where *i* (resp. *j*) has mutated, and compute *G*′ = max (*G*_*i*_, *G*_*j*_). Pairs (*i*, *j*) are binned by the value of *G*′ for the violin plot representation. Two Pearson correlation coefficients are shown: the first one, *ρ*, is obtained when considering all coupling values, and the second one, *ρ*_*M*_, is obtained when considering only the median of the coupling values. Note that no binning on *G*′ is employed in the computation of these Pearson correlations. Couplings were inferred on 100 realizations of the data generation in each case, and aggregated together.

How well does the insight gained from our minimal model apply to natural data? To address this question, we generate synthetic data directly using DCA Potts models inferred on natural MSAs, and phylogenies inferred on the same MSAs. Specifically, we construct three different datasets for each of the four protein families in electronic supplementary material, table S1: natural sequences, equilibrium sequences from the inferred DCA model, and sequences generated using the same inferred DCA model, but along an inferred phylogenetic tree (see Models and methods). These synthetic datasets are closer to natural data than those generated from our minimal model, but retain the advantage that we know when in the phylogeny each mutation occurred. Figure 6 shows the effect of phylogenetic correlations on couplings inferred by MI and plmDCA with or without APC on this data. The main conclusion from our minimal model still holds: higher coevolution scores are obtained for pairs of sites that mutate early in the phylogeny, for both MI and plmDCA without APC. The magnitude of this trend depends on the protein family considered, which may be due to the diversity of their phylogenies (note that the ratio of the effective number of sequences to the actual number of sequences takes diverse values among these families, see electronic supplementary material, table S1). How do phylogenetic corrections impact this trend? We first compare the effects of different phylogenetic corrections (APC, phylogenetic reweighting) on the performance of contact inference on natural sequences (see electronic supplementary material, table S2). While both corrections enhance performance and their combination is best, APC improves contact prediction substantially more than phylogenetic reweighting. Consistently, figure 6 shows that APC strongly impacts the dependence of coupling strength on phylogeny, as we then generally obtain smaller absolute Pearson correlations of the coevolution scores with the earliest mutation, especially for plmDCA. The overall trend is even reversed by APC for MI, as APC-corrected MI scores are lower for pairs of sites that mutate early in the phylogeny.

The contact maps for each of these natural and realistic synthetic datasets are shown in electronic supplementary material, figures S12 and S13, using MI and plmDCA, respectively, and the TP fractions with or without phylogenetic corrections (phylogenetic reweighting and/or APC) are shown in electronic supplementary material, table S3. Note that here, contrarily to our minimal model, the couplings of the inferred DCA models contain some phylogenetic contributions. However, it is fair to compare the equilibrium (independent) and phylogenetic datasets, using the contact map from the bmDCA model (see Models and methods and [41]) as ground truth. We find that phylogeny deteriorates contact prediction performance in these datasets, corroborating the findings from our minimal model. Besides, electronic supplementary material, table S3 confirms that phylogenetic corrections almost always improve the performance of contact prediction. We also find that the number of FP predicted contacts having shortest path length *L* = 2, i.e. indirect correlations, is smaller for plmDCA than for MI, consistently with electronic supplementary material, figure S7 (see electronic supplementary material, table S3). Finally, and perhaps most interestingly, the number of these FPs with *L* = 2 is often smaller in the dataset generated with phylogeny than in the equilibrium one, even though the TP fractions decrease as well, suggesting that most FPs are due to phylogeny (and not to the network of contacts) in the phylogenetic datasets.

## 3. Discussion

Global statistical methods, known as Potts models or DCA, outperform local methods based on covariance or MI at the task of unsupervised prediction of structural contacts from protein sequence data [9–11]. Perhaps surprisingly, these global methods do not outperform local methods as clearly when applied to synthetic data generated independently at equilibrium from DCA models [1]. A usual justification for the success of global methods for protein contact prediction is that they allow to disentangle direct and indirect correlations in the data [9], an effect that is present in synthetic data as well, and can be tuned by varying sampling temperature around a ferromagnetic–paramagnetic phase transition [1,38]. An important difference between natural data and synthetic data sampled independently at equilibrium is the fact that homologous natural sequences share the same ancestry, and thus feature correlations due to phylogeny [2,4]. These correlations obscure structural ones in the identification of structural contacts both by local [27] and global statistical methods [4,28–30].

In this context, we investigated the impact of phylogeny on inference performance by generating synthetic data from a minimal model incorporating both structural contacts and phylogeny. We showed that substantial correlations appear between sites that mutate early in the phylogeny, yielding FP contacts. We found that Potts models are substantially more robust to phylogeny than local methods. This result holds whether or not phylogenetic corrections are used. This robustness to phylogeny contributes to explaining the success of global methods. We showed that our findings from the minimal model generalize well to more realistic synthetic data generated from models inferred on natural data.

The resilience of Potts models to phylogenetic correlations can be understood qualitatively in the simple mean-field approximation, where the DCA couplings are the elements of the inverse covariance matrix of the MSA. Indeed, phylogenetic correlations are associated with large eigenvalues of the covariance matrix [2,4], whose overall impact is reduced by inverting the covariance matrix [4].

Our findings illustrate the crucial impact of data structure on inference performance. Protein MSAs are highly structured datasets, with correlations coming from functional constraints such as structural contacts, as well as from phylogeny, and disentangling these signals is fundamentally difficult [36]. The fact that Potts models succeed better than local methods at this task sheds light on the reasons for their performance for contact inference from natural MSAs. Very recently, protein language models based on MSAs, especially MSA Transformer, have outperformed Potts models at unsupervised contact prediction [42]. We showed in another work that they are even more resilient to phylogenetic noise than Potts model [43]. Taken together, the present results and those of Lupo *et al.* [43] demonstrate the crucial importance of disentangling phylogenetic correlations from functional ones for contact prediction performance. The ability to partly disentangle these correlations is one of the reasons for the success of Potts models, as we showed here, and also of protein language models, as we showed in [43]. While phylogenetic correlations are an issue for structure prediction, they are nevertheless an important and helpful signal for the inference of interaction partners among the paralogs of two protein families [39,44,45]. They are thus a double-edged sword for inference from MSAs. It is our hope that a better understanding of the impact of data structure on inference performance will help developing efficient and interpretable methods in the future.

## 4. Models and methods

### 4.1. Synthetic data generation

#### 4.1.1. General approach

We generate synthetic sequences with fixed structural constraints, modelling those that would exist in a given protein family that needs to maintain structure and function. We consider two types of datasets. The first one is generated at equilibrium under these structural constraints. The second one has an evolutionary history given by a simple phylogeny, and we assume that the same structural constraints have existed throughout this evolutionary history.

To model structural constraints, we use an Erdös–Rényi random graph with 200 nodes and edge probability *q* = 0.02. This graph is held fixed because it models the structural constraints of a given protein family. Each node corresponds then to an amino acid site, and each edge to a structural contact. This number of sites and this density of contacts are in the range of those observed in natural proteins (see electronic supplementary material, table S1). However, because contact density depends on the threshold used to define contacts, it is interesting to consider larger values of *q* (see electronic supplementary material, table S1). In electronic supplementary material, section S4, we also consider a graph with *q* = 0.2 [1]. For simplicity, we model amino acids as Ising spins, i.e. binary variables. We consider that couplings between sites exist on the graph edges, and within our minimal model, we take a unique ferromagnetic coupling value for them (set to one). The corresponding Hamiltonian reads

4.1 $$H(\sigma )=-\sum_{i=1}^{\ell }h_{i}\sigma_{i}-\sum_{\;j=1}^{\ell }\sum_{i=1}^{\;j-1}J_{ij}\sigma_{i}\sigma_{j}=-\sum_{(i,j)\in E}\sigma_{i}\sigma_{j},$$

where a sequence $\sigma =(\sigma_{1},..,\sigma_{\ell })\in {\left\{\pm 1\right\}}^{\ell }$ gives the state of each Ising spin, while ℓ = 200 is the sequence length, and E is the set of edges in the Erdös–Rényi graph. The parameter *h*_*i*_ represents the field at site *i* (here *h*_*i*_ = 0 for all *i*) and *J*_*ij*_ the coupling between sites *i* and *j* (*J*_*ij*_ = 1 if (i,j)∈E, and 0 otherwise).

#### 4.1.2. Generating sequences with structural constraints only

Independent equilibrium sequences are generated by Metropolis Monte Carlo sampling using the Hamiltonian in equation (4.1), which models structural constraints. Each sequence is randomly and independently initialized. Then, moves are proposed, using one of two different methods, either by flipping single spins or clusters of spins (Wolff cluster algorithm) [46]. In both methods, these moves are accepted or rejected according to the Metropolis criterion, with probability

4.2 $$p=\text{min}\left[1,\exp\left(-\frac{\Delta H}{T}\right)\right],$$

where Δ*H* is the difference in the value of *H* after and before the flip, and *T* is the Monte Carlo sampling temperature. This process is iterated until a certain number of moves are accepted. We choose this number so that equilibrium is reached, which can be checked by the saturation of the absolute magnetization (see electronic supplementary material, figure S1). Note that the decorrelation time of absolute magnetization is similar to the magnetization saturation time.

#### 4.1.3. Generating sequences with structural constraints and phylogeny

To include phylogeny in the sequence generation process, we start from an equilibrium ancestor sequence produced as explained above at a given temperature *T*. Then, this sequence is evolved by successive duplication and mutation events (‘generations’) on a binary branching tree, for a total of G=11 generations (see figure 1). Mutations are modelled as single spin flips, and each of them is accepted according to the Metropolis criterion at temperature *T* (see equation (4.2)) with the Hamiltonian in equation (4.1) accounting for structural constraints. We perform a fixed number *μ* of accepted mutations on each branch of the phylogenetic tree. The sequences at the leaves of the phylogenetic tree provide a dataset of $2^{G}=2^{11}=2048$ sequences.

Because we start from an ancestral equilibrium sequence, and then employ the Metropolis criterion at the same temperature *T*, all sequences in the phylogeny are equilibrium sequences. Thus, these sequences comprise correlations from the structural constraints, as without phylogeny. Their relatedness adds extra correlations that we call phylogenetic correlations.

#### 4.1.4. Generating more realistic sequences

To extend our study to more realistic data, we generated sequences from Potts models inferred on natural sequence data. Specifically, natural sequences (Pfam Full MSAs [47]) were retrieved for four different protein families (PF00072, PF00512, PF00595, PF02518). Next, a bmDCA Potts model [41] was inferred on each of these natural MSAs. Indeed, bmDCA has a good generative power, demonstrated experimentally [20], and thus allows to generate new sequences by Metropolis MCMC sampling [41]. (Adaptive cluster expansion, ACE [48,49], an alternative generative method, proved too computationally demanding for our analysis.) We used this procedure to produce an equilibrium dataset of sequences from each of the inferred Potts models. We also generated another set of sequences that contains phylogeny using each of these Potts models [39,43]. For this, we first inferred a phylogenetic tree using FastTree [50] from the natural sequences. At its (arbitrary) root, we place a sequence generated at equilibrium by bmDCA. Then, the sequence evolves according to the inferred phylogeny, where proposed mutations are accepted using the same Metropolis criterion as for the equilibrium data. All sequences at the leaves are collected to form the phylogenetic dataset. Thus, three datasets are available for each protein family: a natural dataset, an equilibrium synthetic dataset and a phylogenetic synthetic dataset.

### 4.2. Inferring contacts from sequences

#### 4.2.1. General approach

We employ methods developed for contact prediction in natural proteins to infer the edges (or contacts) in the graph, and investigate how they are affected by phylogeny. In this spirit, the sequences (either a set of equilibrium sequences generated at a given *T*, or all sequences at the leaves of a given phylogenetic tree) are put together to form a MSA, i.e. a matrix where each row is a sequence and each column is a site. Note that here, there is no alignment issue because each node of the graph is well identified. We compare four different inference methods, which attribute a score to each pair of sites (nodes) in the Erdös–Rényi graph. Top-scoring pairs are predicted contacts. The first two inference methods are local methods, based, respectively, on covariance (*C*) and on MI [27]. The other two methods are global methods, which consist in learning a maximum entropy model consistent with the one- and two-site frequencies observed in the data. These pairwise maximum entropy models, also known as Potts models, or DCA [9], can be approximately inferred in various ways. We consider two widely used versions, namely mean-field DCA (mfDCA) [10,11], which is the simplest one, and plmDCA [32,37], which is the state-of-the-art contact prediction method based on Potts models.

All these methods start from the single-site frequencies of each state *σ*_*i*_ at each site (or column in the MSA) *i* ∈ {1, …, ℓ}, denoted by *f*_*i*_(*σ*_*i*_), and the two-site frequencies *f*_*ij*_(*σ*_*i*_, *σ*_*j*_). In addition, MI and DCA methods employ regularization schemes. First, a pseudocount is added in the computation of the frequencies for MI [44,51] and for mfDCA [10,11], preventing divergences when computing MI or when inverting the covariance matrix in mfDCA. The pseudocount-corrected frequencies read

4.3 $${\tilde{f}}_{i}(\sigma_{i})=\frac{\lambda }{2}+(1-\lambda )f_{i}(\sigma_{i}),$$

4.4 $${\tilde{f}}_{ij}(\sigma_{i},\sigma_{j})=\frac{\lambda }{4}+(1-\lambda )f_{ij}(\sigma_{i},\sigma_{j})\;\mathrm{for}\;i\neq j$$

4.5 $$\mathrm{and}\;\;\;{\tilde{f}}_{ii}(\sigma_{i},\sigma_{j})=\delta_{\sigma_{i}\sigma_{j}}{\tilde{f}}_{i}(\sigma_{i}),$$

where $\delta_{\sigma_{i}\sigma_{j}}=1$ for *σ*_*i*_ = *σ*_*j*_ and 0 otherwise. This correction improves contact prediction from protein sequences by mfDCA [10,11]. In plmDCA, no pseudocount is employed, but an *L*^2^ norm regularization is used, with strengths *λ*_*h*_ on fields and *λ*_*J*_ on couplings [32,37]. Unless otherwise specified, we use the standard values for all these regularization parameters, namely *λ* = 0.01 for MI, *λ* = 0.5 for mfDCA and *λ*_*J*_ = *λ*_*h*_ = 0.01 for plmDCA. An analysis of the impact of pseudocount and regularization strength on contact prediction by mfDCA and plmDCA is provided in electronic supplementary material, figure S6. We compare results with and without APC [27], but we do not employ phylogenetic reweighting [9] in the analysis of our minimal model, because the phylogeny is balanced in that case, which would yield threshold effects in this correction. Besides, we find in our analysis of natural and more realistic data that APC yields a more substantial performance increase than phylogenetic reweighting.

#### 4.2.2. Covariance

The covariance between two sites *i* and *j* reads *C*_*ij*_ = 〈*σ*_*i*_*σ*_*j*_〉 − 〈*σ*_*i*_〉〈*σ*_*j*_〉, where 〈 · 〉 denotes a mean across all sequences of the MSA. While this standard definition can be employed directly with Ising spins, the state of a site in a protein sequence is a categorical random variable with 21 possible states (the 20 natural amino acids and the gap), and therefore frequencies are generally employed instead of means. The two descriptions are equivalent for Ising spins. Indeed,

4.6 $$\langle \sigma_{i}\rangle =f_{i}(1)-f_{i}(-1)=2f_{i}(1)-1$$

and

4.7 $$\begin{array}{rl} \left\langle \sigma_{i}\sigma_{j}\right\rangle & =f_{ij}(1,1)-f_{ij}(1,-1)-f_{ij}(-1,1)+f_{ij}(-1,-1)\\ & \;=1-2f_{i}(1)-2f_{j}(1)+4f_{ij}(1,1), \end{array}$$

where we have employed the marginalization relationships, *f*_*i*_(*σ*) = *f*_*ij*_(*σ*, 1) + *f*_*ij*_(*σ*, − 1) and *f*_*j*_(*σ*) = *f*_*ij*_(1, *σ*) + *f*_*ij*_( − 1, *σ*) for *σ* ∈ { − 1, 1}, and the normalization relationship *f*_*i*_(1) + *f*_*i*_( − 1) = 1, which yield *f*_*ij*_(1, − 1) = *f*_*i*_(1) − *f*_*ij*_(1, 1) and *f*_*ij*_( − 1, 1) = *f*_*j*_(1) − *f*_*ij*_(1, 1), as well as *f*_*ij*_( − 1, − 1) = *f*_*i*_( − 1) − *f*_*ij*_( − 1, 1) = 1 − *f*_*i*_(1) − *f*_*j*_(1) + *f*_*ij*_(1, 1). Combining equations (4.6) and (4.7) yields

4.8 $$C_{ij}=\langle \sigma_{i}\sigma_{j}\rangle -\langle \sigma_{i}\rangle \langle \sigma_{j}\rangle =4[f_{ij}(1,1)-f_{i}(1)f_{j}(1)].$$

We employ the absolute value |*C*_*ij*_| of the covariance to score each pair of sites (*i*, *j*).

#### 4.2.3. Mutual information

The MI of each pair of sites (*i*, *j*) is computed as

4.9 $${\mathrm{MI}}_{ij}=\sum_{\sigma_{i},\sigma_{j}}{\tilde{f}}_{ij}(\sigma_{i},\sigma_{j})\log\left(\frac{{\tilde{f}}_{ij}(\sigma_{i},\sigma_{j})}{{\tilde{f}}_{i}(\sigma_{i}){\tilde{f}}_{j}(\sigma_{j})}\right),$$

where ${\tilde{f}}_{i}(\sigma_{i})$ and ${\tilde{f}}_{ij}(\sigma_{i},\sigma_{j})$ are the pseudocount-corrected one- and two-body frequencies (see equations (4.3)–(4.5)). Note that we use frequencies instead of probabilities to estimate MI, and do not correct for finite-size effects [52]. Indeed, we only compare scores computed on datasets with a given size, affected by the same finite-size effects.

#### 4.2.4. Potts models (DCA)

DCA methods aim to infer the pairwise maximum entropy probability distribution of the data, which is the least constrained distribution consistent with the empirically measured one- and two-body frequencies. The probability of a sequence σ has the following form:

4.10 $$P(\sigma )=\frac{\exp[-H(\sigma )]}{Z},$$

where the Hamiltonian *H* is given by equation (4.1), i.e. the Potts model Hamiltonian, and *Z* is a normalization constant (partition function). The mfDCA method provides a simple approximation of the couplings *J*_*ij*_ in the zero-sum (Ising) gauge [10,11] given by ${\hat{J}}_{ij}=-{\left({\tilde{C}}^{-1}\right)}_{ij}$, where $\tilde{C}$ is the covariance matrix computed with the pseudocount-corrected frequencies (see equations (4.3)–(4.5)), whose elements read ${\tilde{C}}_{ij}=(1-\lambda )[C_{ij}+\lambda \langle \sigma_{i}\rangle \langle \sigma_{j}\rangle ]=(1-\lambda )\langle \sigma_{i}\sigma_{j}\rangle -{\left(1-\lambda \right)}^{2}\langle \sigma_{i}\rangle \langle \sigma_{j}\rangle$ for *i* ≠ *j* and ${\tilde{C}}_{ii}={\left(1-\lambda \right)}^{2}C_{ii}+\lambda (2-\lambda )={\left(1-\lambda \right)}^{2}$ $\left(1-{\left\langle \sigma_{i}\right\rangle }^{2})\;+\lambda (2-\lambda \right)$. Finally, the plmDCA method provides another estimation ${\hat{J}}_{ij}$ of the couplings *J*_*ij*_ by maximizing the pseudolikelihood of equation (4.10). We also use the zero-sum gauge for plmDCA [32,37], and we employ the absolute value $\left|{\hat{J}}_{ij}\right|$ of the inferred coupling to score each pair of sites (*i*, *j*). With natural sequences and realistic synthetic sequences with 21 states, the Frobenius norm of the 21 × 21 matrix of coupling values ${\hat{J}}_{ij}(\alpha ,\beta )$ is used to obtain one score per pair of sites [10,11] and it is denoted by $\left\|{\hat{J}}_{ij}\right\|$.

#### 4.2.5. Evaluating performance

We assess performance via the fraction of correctly identified contacts among the *N*_contacts_ top-scoring pairs of sites, where *N*_contacts_ = 413 is the number of actual contacts, i.e. of edges in the Erdös–Rényi random graph used to generate the data. We refer to this fraction as the true positive fraction (TP fraction). Because we evaluate it for a number of predicted contacts equal to the number of actual contacts, we have TP + FP = TP + FN = *N*_contacts_, where TP denotes the true positives, FP is the false positives and FN is the false negatives. Thus, the TP fraction is equal to the sensitivity, recall, hit rate or true positive rate TPR = TP/(TP + FN), but also to the precision or positive predictive value PPV = TP/(TP + FP).

We employ the TP fraction (or PPV) as a measure of performance because the usual practice for natural sequence data is to predict the top-scoring pairs of sites as contacts. It is also interesting to evaluate how the quality of predictions depends on the number of predicted contacts. For this, one can consider the area under the receiver operating characteristic (AUC). We found very similar conclusions for the AUC as for the TP fraction (see electronic supplementary material, figures S4 and S5).

#### 4.2.6. Extension to more realistic data

Inference on natural and more realistic data is performed using one local method and one global method, respectively, MI and plmDCA. For MI, we employ equation (4.9), using a pseudocount of 0.01, with *σ*_*i*_ ∈ {1, …, 21}. For plmDCA, we use the implementation of the PlmDCA package (https://github.com/pagnani/PlmDCA). Unless otherwise specified, the default values of the parameters of this package are used.

For natural and more realistic data, the number of predicted contacts is taken as *N*_pred_ = 2ℓ, where ℓ is the length of the protein (number of amino acid sites, reported in electronic supplementary material, table S1), and performance is measured as the PPV (or TP fraction) at this number of predicted contacts [10,11].

## Data Availability

All relevant data are included in the manuscript or in the electronic supplementary material [53]. Our code is freely available at https://zenodo.org/record/7503931.
